# Self-Administered Tuberculosis Treatment Outcomes in a Tribal Population on the Indo-Myanmar Border, Nagaland, India

**DOI:** 10.1371/journal.pone.0108186

**Published:** 2014-09-26

**Authors:** Mrinalini Das, Petros Isaakidis, Rahul Shenoy, Rey Anicete, Hemant Kumar Sharma, Imyangluba Ao, Kaikho Osah, Homa Mansoor, Peter Saranchuk, Sunita Abraham

**Affiliations:** 1 Médecins Sans Frontières, Mon district, Nagaland, India; 2 District TB Control Office (RNTCP), Mon district, Nagaland, India; 3 Southern Africa Medical Unit (SAMU), Médecins Sans Frontières, Cape Town, South Africa; Universidad Nacional de La Plata, Argentina

## Abstract

**Background:**

Multiple strategies are being adopted by national tuberculosis (TB) programmes to achieve universal coverage of tuberculosis treatment. However, populations living in ‘hard-to-reach’ areas of north-east India have poor access to health services. Our study aimed to detail treatment outcomes in TB program supported by Médecins Sans Frontières (MSF) and using an alternative model of TB treatment delivery in Mon district, Nagaland, India.

**Methods:**

This was a retrospective cohort study of TB patients, initiated on self-administered therapy (SAT) through Mon District Hospital, Nagaland, India between April 2012 and March 2013.

**Results:**

A total of 238 tuberculosis patients had final TB treatment outcomes during the study period, including 82 and 156 from semi-urban and rural areas respectively. The majority of patients (62%, 147/238) were suffering from pulmonary, smear-positive tuberculosis. Overall, 74% of patients (175/238) had successful outcomes, being cured or having completed their treatment. Females (81%), pulmonary TB patients (75%) and those on a Category I regimen (79%) had better treatment success rates than males (67%), extra-pulmonary TB patients (62%) and patients on a Category II regimen (61%). The univariate and bivariate analyses found age, sex and TB treatment regimen significantly associated with unsuccessful TB treatment outcomes (defined as death, loss-to-follow-up and failure). However, only older age showed significance in a multivariate binary logistic regression model.

**Conclusion:**

Our study suggests that self-administered TB treatment is feasible for patients living in areas with limited or no access to health services. The relatively low number of patients with adverse outcomes suggests that SAT models are safe; other advantages include the need for fewer resources and less frequent movements by patients. National TB programmes should consider allowing SAT strategies for delivery of TB treatment to ‘hard-to-reach’ populations, which could in turn help to achieve universal coverage and contribute to global TB elimination by 2050.

## Introduction

India’s enormous tuberculosis (TB) burden, with an estimated 2.3 million cases annually, contributes more than a quarter of the global TB burden [1]. Multiple strategies are being adopted by the Revised National Tuberculosis Control Programme (RNTCP) to achieve universal coverage of tuberculosis diagnosis and treatment. However, rural populations such as those living in the hilly terrain of Nagaland have limited access to health structures due to “poor road conditions and lack of basic communication services” [2], which may also hinder completion of TB treatment. The district of Mon in the state of Nagaland lies in a ‘hard-to-reach’ area near the Indo-Myanmar border, where several barriers to universal tuberculosis program coverage exist.

India is working towards the World Health Organization (WHO) target of an 85% treatment success rate among all tuberculosis patients [3], including populations in remote and ‘hard-to-reach’ areas. The RNTCP has been present in Nagaland since 2001 [4]; the entire state is now covered under the programme, with a total of 3,334 TB patients initiated on treatment in 2013 [5]. Nagaland and nearby areas in north-eastern India have a significant proportion of drug users and the proportion of TB patients co-infected with HIV is 7 percent [5], which may influence tuberculosis treatment outcomes in such co-infected patients. Even though, Nagaland state is covered under the RNTCP, some pockets still need additional support in order to achieve universal access to TB care and treatment.

“Directly observed treatment, short course (DOTS)” is necessary for the success of a TB control programme, according to the RNTCP [6]. Direct observation helps to ensure treatment compliance and limits development of drug resistance [3]. Some studies have reported on successful treatment outcomes in TB patients that received their complete treatment under DOTS [7]–[9]. However, supervised treatment needs to be adapted to different settings that are convenient to patients, and there must be flexibility in how it is applied [10], whether this is in a health facility, at the workplace, in the community or at home. Human resource issues may compromise a TB programme that relies solely on DOT; for example, many regions in the north-east of India, including Nagaland, do not have enough DOTS providers to allow supervised treatment of all TB patients. Moreover, a Cochrane systematic review and meta-analysis detailed no difference in cure rates between tuberculosis patients taking self-administered therapy (SAT) or receiving DOTS [11], and concluded that DOT did not improve outcomes [10]. For these two reasons, the option of self-administered therapy (SAT) was offered to TB patients, along with strong adherence support measures in the TB programme in Mon district, Nagaland.

Since 2010, Médecins Sans Frontières (MSF)/Doctors Without Borders has been supporting Mon District Hospital, Nagaland, especially with secondary health services. From 2012, MSF provided support to the Mon TB programme including supervision of diagnostic facilities and adherence counseling. Our study aims to detail treatment outcomes of patients receiving self-administered treatment for tuberculosis, in this ‘hard-to-reach’ area on the Indo-Myanmar border.

## Methods

### Ethics

The study satisfied the criteria for reports using routinely collected programmatic data set by the Médecins Sans Frontières independent Ethics Review Board in Geneva, Switzerland. As this was a study of routinely collected monitoring data, informed consent from the patients was not obtained. The named ethics committee specifically approved the study and waived the need for consent.

### Study design

This was a retrospective, observational cohort study.

### Setting and study population

Nagaland is one of the seven north-eastern states of India sharing an international border with Myanmar on the east, while other sides are bounded by Assam, Manipur and Arunachal Pradesh. Mon is one of 11 districts in Nagaland and the farthest one from the capital of the state (Kohima). The district is composed of 14 sub-districts, 2 towns and 131 villages and is inhabited by the tribal population known as ‘Konyak Nagas’, which are- one of the major tribes of Nagaland [2]. The Designated Microscopy Centre (DMC) located in Mon District Hospital provides TB treatment to the people of Mon district plus those from Myanmar near the border areas.

The study population consisted of drug-susceptible tuberculosis patients, initiated on self-administered TB treatment (SAT) in Mon DMC between April 2012 and March 2013 and for whom treatment outcomes were available. Patients registered for care during the study period but who were still on treatment at the end of October 2013 were excluded.

### Tuberculosis treatment delivery

Patients suffering from active tuberculosis in semi-urban Mon town and nearby villages received treatment under the Mon DMC. TB patients on both Category I and Category II treatment regimens were given anti-TB medication three times per week based on RNTCP guidelines [12]. After diagnosis, patients received education about TB and its management, plus adherence counseling by counselors in Mon DMC, with special focus on alcohol and drug abusers.

For the first two weeks, patients received directly observed treatment at Mon DMC. Patients from rural areas had to stay in Mon town with relatives or got admitted in hospital for the two weeks. After two weeks, trained counselors would determine if patients were able to initiate self-administered therapy. The hilly terrain, almost non-existent public transport, poor road connectivity and low number of DOT providers were identified as major challenges for patients to access DMC for supervised treatment administration; patients with such challenges were initiated on self-administered therapy (SAT) for their treatment.

The patients were given medicines for two months (6 weeks medication and 2 weeks buffer) and advised to return after six weeks for treatment follow-up. During each follow-up, smear examinations were carried out for all patients. Other blood evaluations, including kidney and liver function tests, were carried out for patients in need. Adherence levels were evaluated by the empty blister packs and marked calendars brought by patients during follow-up visits. Patients were reminded about their follow-up appointments, one week before their appointment, through mobile phone calls and messages sent to co-villagers.

### Data collection and analysis

Demographic and clinical information were systematically recorded in patient files. The clinical data being routinely collected for each patient, including treatment and laboratory data, were entered into an electronic database. A full time data manager routinely supervised data entry for accuracy and completeness. Data from all study participants initiated on TB treatment between April 2012 and March 2013 and having treatment outcomes were included in the analyses. For the study, patients receiving tuberculosis treatment were categorized as ‘semi-urban’ (residents of the town where Mon DMC was situated) and ‘rural’ (patients from nearby and distant villages). These rural patients took 3–4 hours on an average to reach the DMC via foot/private transport and stayed overnight in Mon town at the home of their relatives during treatment. Successful outcomes were defined as bacteriologically confirmed cure or treatment completion, while adverse outcomes were defined as death, failure or loss to follow up.

Descriptive statistics were used and comparisons between subgroups were made by using t-test and/or chi-square test as appropriate. To explore associations between clinical and demographic factors and unfavorable TB treatment outcomes, bivariate and multivariate analyses were performed using binary logistic regression models. Data analysis was conducted with SPSS (Release 20, 2011).

## Results

### Patient characteristics

A total of 238 patients received self-administered TB treatment (SAT) between April 2012 and March 2013 (Table 1). About half of the study population belonged to the “young and productive age group” (aged 16–35 years), while 20% patients were children aged less than 16 years. The majority (56%) of the patients were male. The ratio of pulmonary smear-positive: pulmonary smear-negative: extra-pulmonary TB was 147∶62∶29. Among the study population with recorded HIV status, 0.14% (3/222) TB patients were co-infected with HIV. The majority (72%) of patients were receiving Category I treatment [12] for tuberculosis compared to patients on Category II treatment for tuberculosis (28%).

**Table 1:** Demographic and clinical characteristics of patients receiving self-administered tuberculosis treatment (SAT) in Mon district, Nagaland, India, 2012–2013.

Characteristics	Tuberculosis patients on treatment (N = 238)
	**n (%)**
**Age** (years)	
0–15	47 (19.7)
16–25	71 (29.8)
26–35	48 (20.2)
36 and above	72 (30.3)
**Sex**	
Male	132 (55.5)
Female	106 (44.5)
**Residence**	
Rural	156 (65.5)
Semi-urban	82 (34.5)
**TB site**	
Pulmonary, smear-positive	147 (61.8)
Pulmonary, smear-negative	62 (26.1)
Extra-pulmonary	29 (12.2)
**TB treatment regimen**	
Category I	172 (72.3)
Category II	66 (27.7)

Among these 238 patients receiving self-administered TB treatment, 82 patients were from a ‘semi-urban’ area (i.e. Mon town where the DMC was situated) while the other 156 patients were from a ‘rural’ area. The demographic and clinical characteristics of these two groups were compared (Table 2) using univariate and bivariate analysis. The results of the analysis showed that age, sex, TB site and treatment regimen was not statistically different between the groups, thus confirming that the patients in the ‘semi-urban’ and ‘rural’ groups were similar.

**Table 2:** Demographic and clinical characteristics of ‘Semi-urban’ and ‘Rural’ patients receiving self-administered tuberculosis treatment (SAT) in Mon district, Nagaland, India, 2012–2013.

Characteristics	‘Semi-urban’ patients (N = 82) n (%)	‘Rural’ patients (N = 156) n (%)	Chi-square/t-test (p-value)
**Age** (years, median, IQR)	25.0 (18.0–35.3)	28.0 (17.3–40.0)	0.80 (0.43)
**Sex**			
Male	48 (58.5)	84 (53.8)	0.48 (0.49)
Female	34 (41.5)	72 (46.2)	
**TB site**			
Pulmonary, smear-positive	51 (62.2)	96 (61.5)	0.80 (0.67)
Pulmonary, smear-negative	23 (28.0)	39 (25.0)	
Extra-pulmonary	8 (9.8)	21 (13.5)	
**TB treatment regimen**			
Category I	59 (72.0)	113 (72.4)	0.01 (0.94)
Category II	23 (28.0)	43 (27.6)	

IQR: Interquartile range.

### Treatment Outcomes

Overall, 74% (175/238) of the entire study population (i.e. including patients from semi-urban and rural) were cured or had completed treatment (Table 3). Females had a higher proportion (81%) of successful treatment outcomes compared to males (67%). The pulmonary TB patients had better treatment success rates (75%) than extra-pulmonary (EPTB) patients (62%). Patients on category I regimen had more successful treatment outcomes than patients on category II (79% versus 61%).

**Table 3:** Demographic and clinical factors associated with unsuccessful treatment outcomes among patients receiving self-administered TB treatment (SAT) in Mon district, Nagaland, India, 2012–2013.

ExplanatoryVariable	Patients with unsuccessfuloutcome† (N = 63), n (%)	Patients with successfuloutcome† (N = 175), n (%)	Chi-square/t-test(p-value)	aOR^a^(95% CI)
**Age** (years, median, IQR)	36 (22–50)	24 (16–35)	3.81 (<0.01)	1.03 (1.01–1.05)
**Sex**				
Male	43 (32.6)	89 (67.4)	5.68 (0.02)	1.50 (0.78–2.86)
Female	20 (18.9)	86 (81.1)		
**Residence**				
Semi-urban	24 (29.3)	58 (70.7)	0.50 (0.48)	-
Rural	39 (25.0)	117 (75.0)		
**TB site**				
Pulmonary	52 (24.9)	157 (75.1)	2.23 (0.14)	-
Extra-pulmonary	11 (37.9)	18 (62.1)		
**TB treatment regimen**				
Category I	37 (21.5)	135 (78.5)	7.84 (0.01)	
Category II	26 (39.4)	40 (60.6)		1.81 (0.95–3.47)

† Row percentage in parenthesis.

IQR: Inter-quartile range.

a aOR: adjusted Odds Ratios (calculated by binary logistic regression).

To understand the factors associated with unsuccessful TB treatment outcome in the study population, the demographic and clinical factors were assessed for association with unsuccessful outcome. The univariate and bivariate analyses found that age, sex and TB treatment regimen were significantly related to an unsuccessful TB treatment outcome. However, none of the factors other than older age (adjusted Odds Ratio: 1.03, 95% Confidence Intervals (CI): 1.01–1.05) were associated with unsuccessful TB treatment outcome in a multivariate binary logistic regression model.

## Discussion

Our study, the first on tuberculosis treatment outcomes from this district, suggests that self-administered TB treatment (SAT), is an alternative model for delivery of TB treatment to people living in areas with limited or no access to health services.

The results demonstrate that successful TB outcomes may be achieved irrespective of the distance between a person’s home and the nearest functional TB facility. The majority of study participants from rural areas, who otherwise would have had to travel an average of three hours from distant villages to reach Mon DMC, were instead able to successfully complete their TB treatment with far less movements. SAT models of care and ‘TB villages’ have been adopted in various settings for the management of TB in mobile populations [13], [14]. Low numbers of patients with adverse outcomes in our study and another by Khogali et al [13] suggest that modified models of treatment delivery can be considered for ‘hard-to-reach’ populations. Not only do they require little direct supervision, but such alternative models can reduce resources required, frequency of movements and stigma felt by patients from involuntarily disclosure of their TB status, as a result of multiple visits to a health facility per week to receive DOT.

The adult (15–49 years) HIV prevalence in state of Nagaland is 0.73 percent [15]. Despite the low prevalence, the areas in the north-eastern states of India constitute 30% of Injecting drug users (IDU) of the country [15], which may influence adherence to tuberculosis treatment [16] and overall treatment outcomes. Appropriate and effective monitoring systems should be devised for patients on SAT models, administered by trained counselors using simple and validated tools [17]. The patients in the study were reminded about their follow-up appointments through mobile phone calls and messages sent to co-villagers. Thus, we must assume that these reminders and other adherence support measures played a role in the successful TB outcomes seen in this cohort. National TB programmes should consider use of trained counselors to improve adherence levels and treatment outcomes of TB patients in other settings.

Literacy status, distance from health structure [18], quality of medications administered, daily or intermittent dosages [19], use of fixed dose combination (FDC) anti-TB therapy versus loose pills [20] and mode of administration have all been discussed in previous studies as potential factors associated with TB treatment outcomes. A study by Nackers et al suggested that self-administered therapy (SAT) with FDC pills may help in achieving appropriate adherence to treatment [17]. Although we were not able to provide FDCs to study population, as these were not readily available through the national TB programme, we believe that the use of FDCs would have simplified the TB management, including counseling sessions for the patients.

The treatment success rate among extra-pulmonary TB (EPTB) patients in our study are alarmingly lower than the WHO target of 85%, which may indicate late arrival and/or delayed diagnosis and poor pre-treatment clinical status of such patients. Although the proportion of study population with HIV/TB co-infection was small, more than one-third of our cohort was suffering from either smear-negative TB or EPTB; this finding needs further attention. Better diagnostic tools are required to allow for prompt diagnosis and treatment of EPTB cases.

About half of our patients belonged to ‘young and productive’ age group and one-fifth were children suffering from TB. We have made several efforts to provide tailored adherence counseling sessions, TB regimens and treatment monitoring tools for these vulnerable populations. Data from a drug-resistant TB programme that cared for a cohort of HIV-infected adolescents in Mumbai showed extremely poor outcomes [21]. The needs of adolescents and pediatric patients with all forms of TB are disturbingly neglected and largely unmet. More patient-centered, decentralized approaches that include use of patient self-help groups, family member as DOT provider or community participation should be considered by TB programmes as opposed to a “one-size-fits-all” strategy.

The strength of this study was that we were able to examine the impact of a self-administered therapy model of care in a remote district with semi-urban and rural areas. This study reflected the operational reality of piloting an alternative to the DOT model of TB treatment, in a geographic location under a national programme that promotes a DOT model but with very limited resources to support it. We were able to achieve promising results due to continuous collaboration with the Mon District Hospital management committee and the national TB program. Our results were similar for patients in both semi-urban and rural areas, showing feasibility of the SAT model in developed towns and remote villages. However, the study findings are limited to patients registered in one particular setting and may not be generalizable to other parts of the country. We were not able to trace the lost-to-follow-up patients who lived far from Mon. Despite these limitations, we believe that this study documents an innovative model of care for TB patients living in ‘hard-to-reach’ areas.

## Conclusion

In settings where access to TB health services is limited or not existing, an alternative to the DOTS strategy for TB, such as self-administered treatment together with adherence support measures, could allow for successful TB treatment outcomes. National TB programmes may consider similar innovative models of care for tuberculosis treatment in order to meet the needs of ‘hard-to-reach’ populations, as these may help to achieve universal coverage of TB treatment, and ultimately reduce the burden of TB in such settings.
